# A Single Nucleotide Polymorphism in the *FADS1* Gene is Associated with Plasma Fatty Acid and Lipid Profiles and Might Explain Gender Difference in Body Fat Distribution

**DOI:** 10.1186/s12944-017-0459-9

**Published:** 2017-03-31

**Authors:** Huilan Guo, Lichao Zhang, Chaonan Zhu, Fei Yang, Shanshan Wang, Shankuan Zhu, Xiaoguang Ma

**Affiliations:** 1 Department of Nutrition and Food Hygiene, School of Public Health, School of Medicine, Zhejiang University, 866 Yu-hang-tang Road, Hangzhou, Zhejiang 310058 China; 2 Chronic Disease Research Institute, School of Public Health, School of Medicine, Zhejiang University, 866 Yu-hang-tang Road, Hangzhou, Zhejiang 310058 China; 3 Department of Public Health, Shandong Provincial Hospital affiliated to Shandong University, Jinan, Shandong China

**Keywords:** SNP, genotype, desaturase activity, lipid profiles, fat mass, fatty acid, fat distribution

## Abstract

**Background:**

Genotyping of the rs174547 polymorphism in the fatty acid desaturase 1 gene (*FADS1*) shows that it is associated with the FA composition of plasma phospholipids and lipid metabolic indices among several ethnic groups. However, this association requires further confirmation in the Chinese population, and little is known about the effect of polymorphisms in fatty acid-related genes on body fat distribution.

**Methods:**

Anthropometric measurements of 951 Chinese adults aged 18–79 were obtained and body fat distribution was estimated using dual-energy X-ray absorptiometry. The FA composition of plasma phospholipids was measured by gas chromatography. Multiple linear regression assessed whether the rs174547 genotype was associated with FA composition, body fat distribution, and metabolic traits in additive, dominant, and recessive models.

**Results:**

The rs174547 C minor allele was associated with a higher proportion of linoleic acid, lower arachidonic acid and docosahexaenoic acid, as well as lower delta-6-desaturase and delta-5-desaturase activity. Female C allele carriers had lower android fat percentages and lower levels of low-density lipoprotein-cholesterol, while male C allele carriers had lower gynoid fat percentages and higher triglyceride after adjusting for age, income, BMI, behavioral risk factors, and regional fat percentages.

**Conclusion:**

An association of *FADS1* rs174547 with the FA composition of plasma phospholipids was identified among this Chinese adult population. The association with body fat distribution and lipid metabolic indices differed between men and women, which might explain sexual differences in body fat distribution and lipid metabolism.

## Background

Previous studies have identified associations between variations in genes encoding fatty acid desaturase 1 (*FADS1*) and 2 (*FADS2*) with changes in plasma FA profiles and altered desaturase activity [1–4]. For instance, *FADS1* rs174547, a T/C SNP in intron 9 of *FADS1*, was reported to be associated with arachidonic acid to linoleic acid (AA:LA) FA ratios in both Caucasians and Asians [5, 6]. Similarly, the association of this locus with fatty acid level was examined among Chinese, but the findings were inconsistent across studies [7, 8].

FA metabolism has been linked to body fat accumulation and obesity [9–12]. However, it is not clear whether the polymorphisms in FA-related genes are associated with obesity and body fat distribution. A previous study of a Chinese population failed to associate *FADS1* rs174547 with BMI [13], but as yet no studies have focused on body composition, especially body fat and its distribution (i.e., body fat percentage and central obesity). Although gender differences have been identified for body fat distribution [14], for example, men tend to accumulate fat in the abdominal region (apple-type obesity) and women in the hip region (pear-type obesity), few genetic variations explain this disparity so far. Moreover, while genome-wide association studies have revealed gene-by-sex interactions for BMI and waist circumference (WC) [15, 16], few studies have explained gender differences in fat distribution measured by issue-specific technology such as dual-energy X-ray absorptiometry (DXA) or computed tomography [17].

*FADS1* rs174547 was shown to be involved in the lipid metabolic pathway which catalyzes the biosynthesis of highly unsaturated FA. Thus, this SNP might impact on human lipid profiles. Previous studies associated the *FADS1* rs174547 genotype with lipids levels in Chinese population. For instance, a Chinese study reported that the CC variant of rs174547 was significantly associated with increased TG and decreased HDL-C [18]. However, the findings were inconsistent in recent studies. In a recent study examining the association between the FADS gene cluster and coronary artery disease and lipids in northern Chinese Han population, no significant associations were found between rs174547 and lipids indicators [19].

This study aimed to examine the effect of *FADS1* rs174547 on plasma FA composition, body composition, body fat indices, and plasma lipid profiles among Chinese adults. We also explored the impact of this SNP on gender differences in body fat distribution.

## Methods

### Study sample

A total of 1029 community residents aged from 18 to 82 years old were recruited voluntarily between November 2008 and May 2009 from two communities in Hangzhou City, a capital provincial city in eastern China. Two communities were chosen for convenience from Xiacheng and Xiaoshan districts of the city. The subjects were recruited voluntarily by posters, flyers, and community leaders. All subjects completed a standardized questionnaire survey, underwent physical anthropometry and body composition measurements, and provided blood samples at the Obesity and Body Composition Research Center of Zhejiang University School of Public Health. In 2014, 67 participants were sampled with stratification on BMI distribution, and the measurement of plasma FA was made using their stored plasma samples. In 2015, after excluding the participants aged <18 or >80 years, the *FADS1* rs174547 genotype was determined in 951 participants (including the 67 participants sampled in 2014) using stored blood samples.

**Fig. 1:**
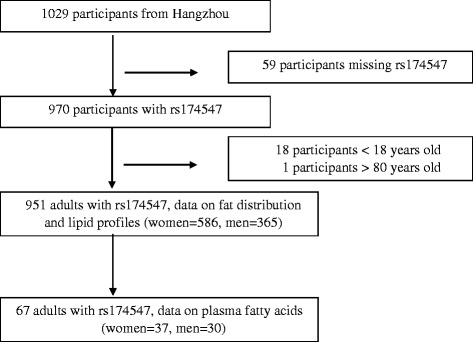
Participant selection and follow-up flowchart

The flow of participants included in the analysis is shown in Fig. 1. Written informed consent was obtained from all participants and the study was approved by the Ethics Committee of the Second Affiliated Hospital of Zhejiang University.

### Measurements

Weight and height were measured with subjects wearing light clothing and without shoes. Body weight was recorded to the nearest 0.1 kg (Detecto, Missouri, USA). Body height was measured with a hypsometer to the nearest 0.1 cm. All values were recorded as the mean of three measures. WC was measured at the approximate midpoint between the lower margin of the last palpable rib and the top of the iliac crest, at minimal respiration. Hip circumference was measured at the maximum circumference of the buttocks with the tape parallel to the floor. Blood pressure was obtained from the right arm of seated participants after a 5-min rest. It was measured twice by the same trained examiners with a mercury sphygmomanometer according to a standard protocol [20]; the mean of the two measurements was used for analysis. BMI was calculated as weight in kilograms divided by height in meters squared.

DXA (GE Lunar Prodigy, WI, USA with software version 11.40.004) was implemented to measure body fat distribution as previously described [21]. The bone mineral density, fat mass, and lean soft tissue mass of the whole body and specific regions of interest (limb, trunk, android region, and gynoid region) were measured. DXA was checked daily against a calibration phantom using the manufacturer’s precision standards of <0.8%. Fat mass percentage (FMP), as an indication of fat distribution, was calculated as regional fat mass kg/body weight in kg*100.

Information on demographics, socioeconomic status, and lifestyle behavior was collected by face-to-face interview using a structured questionnaire, including questions on age, sex, income, smoking status, alcohol consumption, and physical activity. Participants were classified according to their smoking status as nonsmokers or current smokers, and according to their alcohol consumption as non-drinkers, current drinkers, and abstainers. Physical activity was grouped as low, moderate, or heavy based on its frequency and intensity according to the international physical activity questionnaire [22, 23].

### Blood biochemical analysis and DNA extraction

After an overnight fasting, venous blood specimens were collected for biochemical analysis. Fasting plasma glucose (FPG) concentrations were determined using a hexokinase method. Total cholesterol (TC) and TG concentrations were measured using COD-PAP and GPO-PAP methods, and HDL-C and LDL-C were measured using an enzymatic homogeneous assay. The levels of apolipoprotein A1, and apolipoprotein B were determined by immunoturbidimetric assay. All the reagents were obtained from Roche. Then remaining samples were stored at −80 °C for further analysis. Genomic DNA was extracted from 1 to 5 mL whole blood using a commercially available DNA isolation kit (BloodGen Midi Kit, CWBIO, Beijing, China) according to the manufacturer’s protocol between April and May 2015.

### Measurement of plasma phospholipid fatty acid

For the analysis of FA composition, total lipids were extracted from 200 μl plasma using chloroform–methanol (2:1 v:v) according to the Folch method [24]. The homogenates were stilled at 4 °C and subsequently centrifuged at 3000 xg for 5 min, then the lipid-containing chloroform phase was transferred to a new tube and dried under nitrogen gas. The extraction process was repeated with an equal volume of chloroform. The resulting free FAs were methylated by the addition of 14% boron trifluoride in methanol at 100 °C for 1 h. The reaction was stopped by adding distilled water, and the contents were centrifuged at 3000 xg for 5 min and dried under nitrogen gas for phase separation. The lipid phase was evaporated under nitrogen gas, and reconstituted in hexane for analysis. FA methyl esters were separated by gas chromatography using an Agilent 7890A gas chromatograph (Agilent Technologies, Palo Alto, CA). Peaks were identified by comparison with FA methyl ester standards. The levels of plasma FAs were expressed as percent fatty acid composition: palmitic acid (C16:0), stearic acid, palmitoleic acid, oleic acid (OA), LA (C18:2n − 6), α-linolenic acid (C18:3n − 3), γ-linolenic acid (GLA; C18:3n − 6), Docosahexaenoic acid (DHA; C22:6n − 3), AA (C20:4n − 6), and eicosapentaenoic acid (C20:5n − 3). Desaturase activity was estimated using the ratio of the product FA to precursor FA, which has previously been well-established. The Δ6 desaturase (D6D) activity was estimated by dividing the % composition of DGLA by LA, and the Δ5 desaturase (D5D) activity was estimated by dividing the % composition of AA by DGLA [25].

### Statistical analysis

The statistical significance of demographic characteristics was analyzed using the *t*-test for continuous variables or the chi-square test for categorical variables. Pearson’s χ^2^ test was used to examine whether rs174547 genotype frequencies were in Hardy–Weinberg equilibrium (HWE). The associations between *FADS1* rs174547 and plasma phospholipid FAs, body composition indices, and lipid profile indicators were identified by multivariate linear regression models, with each stage of regression analysis performed assuming additive (variant/variant vs. variant/common vs. common/common), dominant (variant/variant + variant/common vs. common/common), or recessive (variant/variant vs. variant/common + common/common) models. The effect value was determined from the model with the lowest *P* value.

For the models identifying associations between *FADS1* rs174547 and plasma phospholipid FAs in 67 participants, the first step was to adjust for sex and age, then to adjust for smoking, drinking, and BMI. For the models identifying associations between rs174547 and anthropometric measurements and lipid indicators in 951 participants, we adjusted the same covariates as above. We further adjusted the fat mass in other body regions to determine whether rs174547 independently influenced regional fat distribution. All analyses in the models of anthropometric measurements and lipid indicators were performed separately in men and women because of the interaction of gender. All statistical analyses were conducted using SAS software version 9.3 (SAS Institute, Cary, NC). A two-tailed value of *P* < 0.05 was considered statistically significant.

## Results

The characteristics and rs174547 genotypes of the FA analysis sample (*n* = 67) are shown in Tables 1 and 2 shows anthropometric measurements and lipid analysis of the whole sample (*n* = 951). For whole sample analysis, men showed higher levels of physical activity, alcohol consumption, and smoking prevalence than women, while no sex difference was observed in age, BMI, or household income. The effect allele of rs174547 was the C allele, which had a frequency of 0.343 and conformed to HWE (*P* = 0.117), with a genotyping success rate of 99.5%.

**Table 1:** Characteristics of the Study Population in the in the fatty acid analysis (*N* = 67)

Variables	Mean ± SD or N (%)			*P* value for gender difference
	Total (*n* = 67)	Men (*n* = 37)	Women (*n* = 30)	
Age (years)	51.6 ± 12.9	50.6 ± 13.2	52.7 ± 12.8	0.259
BMI (kg/m^2^)	23.8 ± 3.1	24.2 ± 3.3	23.1 ± 2.8	0.068
Smoking				<0.001
Non-smoker	43 (64.2)	15 (40.5)	28 (59.5)	
Current smoker	24 (35.8)	22 (93.3)	2 (6.67)	
Alcohol consumption				0.007
Non-drinker	29 (43.3)	10 (27.03)	19 (63.3)	
Current drinker	36 (53.7)	25 (69.4)	11 (36.7)	
Abstainer	2 (3)	2 (5.41)	0 (0)	
Rs174547 genotypes				0.427
TT	26 (38.81)	15 (40.5)	11 (36.7)	
TC	36 (53.73)	18 (48.7)	18 (60.0)	
CC	5 (7.46)	4 (10.8)	1 (3.3)

**Table 2:** Characteristics of the Study Population in anthropometric measurement and lipids analysis (*N* = 951)

Variables	Mean ± SD or N (%)			*P* value for gender difference
	Total (*n* = 951)	Men (*n* = 365)	Women (*n* = 586)	
Age (years)	50.0 ± 13.8	50.5 ± 14.3	49.7 ± 13.5	0.412
BMI (kg/m^2^)	23.5 ± 3.2	23.6 ± 3.3	23.4 ± 3.2	0.336
Smoking				<0.001
Non-smoker	664 (69.9)	98 (26.9)	576 (96.8)	
Current smoker	286 (30.1)	267 (73.1)	19 (3.2)	
Alcohol consumption				<0.001
Non-drinker	493 (51.8)	101 (27.7)	392 (67.0)	
Current drinker	389 (40.9)	235 (64.4)	154 (26.3)	
Abstainer	68 (7.2)	29 (7.9)	39 (6.7)	
Household Income				0.062
< 1w	105 (11.0)	31 (8.5)	74 (12.6)	
1-3w	385 (40.5)	144 (39.5)	241 (41.1)	
3-5w	273 (28.7)	105 (28.8)	168 (28.7)	
> 5w	188 (19.8)	85 (23.3)	103 (17.6)	
Physical activity				
Light	444 (46.7)	192 (52.6)	252 (43.0)	0.004
Moderate	397 (41.7)	143 (39.2)	254 (43.3)	
Heavy	110 (11.6)	30 (8.2)	80 (13.7)	
Rs174547 genotypes				
TT	326 (34.3)	117 (32.1)	209 (35.7)	0.487
TC	437 (45.9)	176 (48.2)	261 (44.5)	
CC	188 (19.8)	72 (19.7)	116 (19.8)	

Correlations between the rs174547 genotype and plasma phospholipid FAs are shown in Table 3. The number of C alleles was positively correlated with the proportion of OA and LA in the additive model and recessive model when adjusting for age and sex. An increase in the number of C alleles was associated with a significantly higher OA level and LA level, and with a significantly lower AA level and D6D in the additive model and recessive model. When including age, sex, smoking, drinking, and BMI in the model, the positive association between the C allele and LA remained significant, while that between the C allele and OA was lost. The C allele was inversely associated with the concentration of AA, DHA, D6D, and D5D after adjusting the covariates.

**Table 3:** The association between rs174547 and plasma fatty acids (*N* = 67)

Fatty acid	Effect (SD)	P value^a^			Effect (SD)	P value^b^		
		Addictive	Dominant	Recessive		Addictive	Dominant	Recessive
Palmitic acid (16:0)	−0.98 (1.09)	0.775	0.904	0.371	−1.13 (1.14)	0.743	0.914	0.324
Stearic acid (18:0)	−0.60 (0.96)	0.865	0.589	0.536	0.32 (0.53)	0.767	0.548	0.650
Palmitoleic acid (16:1)	0.10 (0.06)	0.277	0.591	0.127	0.06 (0.06)	0.476	0.711	0.333
Oleic acid (18:1)	0.40 (0.20)	0.049*	0.057	0.318	0.47 (0.25)	0.074	0.063	0.525
Linoleic acid (18:2)	2.85 (1.42)	0.053	0.181	0.049*	3.62 (1.44)	0.022*	0.128	0.015*
Dihomo-γ-linolenic acid (20:3)	−0.18 (0.25)	0.982	0.727	0.476	−0.37 (0.23)	0.523	0.979	0.116
Arachidonic acid (20:4)	−1.90 (0.41)	<.0001***	<.0001***	0.18	−1.99 (0.41)	<.0001***	<.0001***	0.171
Α-linolenic acid (18:3)	0.17 (0.15)	0.448	0.255	0.711	0.22 (0.15)	0.219	0.147	0.902
Eicosapentaenoic acid (20:5)	−0.26 (0.19)	0.177	0.21	0.438	−0.31 (0.19)	0.114	0.194	0.222
Docosahexaenoic acid (22:6)	−0.1 (0.05)	0.124	0.367	0.06	−0.13 (0.05)	0.04*	0.192	0.020*
Δ6 desaturase	−1.35 (0.38)	0.004**	0.001**	0.723	−1.34 (0.37)	0.005**	0.001**	0.982
Δ5 desaturase	−0.003 (0.008)	0.383	0.696	0.194	−0.03 (0.01)	0.083	0.374	0.017*

^a^Adjusted for age and sex; ^b^Adjusted for age, sex, smoke, drinking, and BMI; *P < 0.05, **P < 0.01, ***P < 0.0001

The associations between the rs174547 genotype and anthropometric indicators among men and women are shown respectively in Tables 4 and 5. In men, no significant association between fat distribution and the rs174547 C allele was observed when only adjusting for age. However, after further adjusting the covariates and BMI, the C allele was negatively correlated with WC, gynoid FMP, and limb FMP. The gynoid FMP remained significantly associated with the C allele in the additive model and dominant model when further adjusting for other regional fat distribution.

**Table 4:** The association between rs174547 and fat distribution in men (*N* = 365)

Indicators	T/T	T/C	C/C		P value^a^		Effect (SD)	P value^b^			Effect (SD)	P value^c^		
	Mean (SD)	Mean (SD)	Mean (SD)	Addictive	Dominant	Recessive		Addictive	Dominant	Recessive		Addictive	Dominant	Recessive
BMI (kg/m^2^)	23.53 (3.50)	23.71 (3.23)	23.70 (3.07)	0.889	0.689	0.822	-	-	-	-	-	-	-	-
Weight (kg)	66.36 (11.06)	66.69 (10.71)	65.91 (9.74)	0.861	0.925	0.670	−0.23 (0.35)	0.518	0.623	0.563	-	-	-	-
Waist circumference (cm)	86.17 (10.53)	85.45 (10.63)	86.02 (10.17)	0.561	0.565	0.719	−1.00 (0.50)	0.124	0.044*	0.704	-	-	-	-
Hip circumference (cm)	91.48 (10.50)	92.30 (6.01)	92.07 (5.24)	0.518	0.375	0.914	0.43 (0.65)	0.526	0.507	0.724	-	-	-	-
Waist-to-height ratio	0.51 (0.08)	0.51 (0.09)	0.52 (0.06)	0.962	0.844	0.882	−0.0006 (0.01)	0.977	0.929	0.958	-	-	-	-
Waist-to-hip ratio	0.93 (0.07)	0.93 (0.13)	0.93 (0.07)	0.509	0.767	0.404	−0.01 (0.01)	0.472	0.675	0.428	-	-	-	-
Android fat mass percent	2.54 (0.93)	2.48 (1.02)	2.54 (0.99)	0.593	0.601	0.736	−0.09 (0.06)	0.236	0.143	0.695	0.04 (0.06)	0.735	0.963	0.509
Gynoid fat mass percent	3.36 (0.98)	3.24 (1.00)	3.13 (1.01)	0.112	0.178	0.212	−0.14 (0.05)	0.010*	0.012*	0.098	−0.10 (0.04)	0.020*	0.040*	0.083
Android/gynoid fat ratio	0.75 (0.19)	0.75 (0.21)	0.80 (0.21)	0.447	0.758	0.318	0.03 (0.02)	0.328	0.719	0.183	-	-	-	-
Total fat mass percent	20.87 (7.19)	20.39 (7.72)	20.53 (7.69)	0.490	0.541	0.609	−0.85 (0.47)	0.111	0.071	0.474	-	-	-	-
Limb fat mass percent	6.9 (2.44)	6.70 (2.44)	6.58 (2.47)	0.287	0.364	0.405	−0.38 (0.18)	0.042*	0.033*	0.259	−0.15 (0.09)	0.089	0.108	0.253
Trunk fat mass percent	13.12 (4.89)	12.85 (5.35)	13.11 (5.26)	0.636	0.664	0.738	−0.45 (0.32)	0.254	0.164	0.693	0.13 (0.29)	0.824	0.974	0.664

^a^Adujsted for age; ^b^Adjusted for age, income, smoking, drinking, physical activity and BMI; ^c^Adjusted for age, income, smoking, drinking, physical activity, B MI, and other regional fat (For android fat mass percent, the gynoid fat mass percent was further adjusted, vice versa; for limb fat mass percent, the trunk fat mass percent was further adjusted, vice versa); *P < 0.05

**Table 5:** The association between rs174547 and fat distribution in women (*N* = 586)

Indicators	T/T	T/C	C/C		P value^a^		Effect (SD)	P value^b^			Effect (SD)	P value^c^		
	Mean (SD)	Mean (SD)	Mean (SD)	Addictive	Dominant	Recessive		Addictive	Dominant	Recessive		Addictive	Dominant	Recessive
BMI (kg/m^2^)	23.60 (3.28)	23.66 (3.06)	22.64 (3.30)	0.026	0.274	0.006**	-	-	-	-	-	-	-	-
Weight (kg)	57.72 (8.75)	58.11 (8.15)	55.11 (8.59)	0.029*	0.424	0.002**	−0.46 (0.42)	0.733	0.71	0.281	-	-	-	-
Waist circumference (cm)	80.54 (9.08)	80.74 (8.45)	77.71 (9.36)	0.017*	0.235	0.003**	−0.47 (0.43)	0.337	0.585	0.271	-	-	-	-
Hip circumference (cm)	92.02 (6.00)	91.89 (5.35)	90.88 (5.94)	0.129	0.327	0.111	0.37 (0.34)	0.63	0.859	0.27	-	-	-	-
Waist-to-height ratio	0.52 (0.06)	0.52 (0.06)	0.50 (0.06)	0.023*	0.181	0.011*	−0.002 (0.002)	0.402	0.367	0.656	-	-	-	-
Waist-to-hip ratio	0.87 (0.06)	0.88 (0.06)	0.85 (0.07)	0.018*	0.357	0.001**	−0.01 (0.01)	0.184	0.696	0.049*	-	-	-	-
Android fat mass percent	3.18 (0.80)	3.12 (0.79)	2.89 (0.87)	0.002**	0.023*	0.005**	−0.09 (0.04)	0.052	0.039*	0.286	−0.09 (0.04)	0.052	0.041*	0.278
Gynoid fat mass percent	5.64 (0.90)	5.59 (0.85)	5.54 (0.97)	0.291	0.442	0.315	−0.02 (0.07)	0.922	0.766	0.856	0.02 (0.08)	0.948	0.900	0.786
Android/gynoid fat ratio	0.57 (0.16)	0.57 (0.16)	0.53 (0.18)	0.038*	0.103	0.068	−0.01 (0.01)	0.231	0.206	0.505	-	-	-	-
Total fat mass percent	32.17 (5.83)	31.94 (5.73)	30.07 (6.48)	0.006**	0.08	0.003**	−0.32 (0.20)	0.118	0.169	0.230	-	-	-	-
Limb fat mass percent	12.80 (2.41)	12.66 (2.39)	12.19 (2.39)	0.040*	0.165	0.037*	−0.10 (0.11)	0.364	0.374	0.557	−0.11 (0.17)	0.522	0.499	0.724
Trunk fat mass percent	18.12 (4.09)	18.05 (4.08)	16.66 (4.65)	0.006**	0.101	0.003**	−0.21 (0.14)	0.14	0.235	0.206	−0.18 (0.14)	0.185	0.299	0.240

^a^Adujsted for age; ^b^Adjusted for age, income, smoking, drinking, physical activity and BMI; ^c^Adjusted for age, income, smoking, drinking, physical activity, BMI, and other regional fat (For android fat mass percent, the gynoid fat mass percent was further adjusted, vice versa; for limb fat mass percent, the trunk fat mass percent was further adjusted, vice versa); *P < 0.05, **P < 0.01

In women, all indicators of body fat distribution except weight, BMI, WC, and weight-to-hip ratio (WHpR) showed a decreasing trend with increasing numbers of C alleles of the respective genotype after adjusting for age. When further adjusting for covariates and BMI, the association of the C allele with WHpR and android FMP remained significantly negatively correlated, while the association with other indicators of fat distribution diminished. When including gynoid FMP in the model, the android FMP remained significantly associated with the C allele in the dominant model.

The associations between the rs174547 genotype and lipid profiles among women and men are shown respectively in Tables 6 and 7. The level of TG was positively correlated with the C allele in the additive model among men when adjusting for covariates and BMI; the association remained significant after further adjusting for android FMP. In women, the C allele was negatively correlated with the concentration of LDL-C in the additive model and dominant model after further adjusting for android FMP.

**Table 6:** Association between rs174547 and lipid profiles in men (*N* = 365)

Indices	Effect (SD)	P value^a^			Effect (SD)	P value^b^			Effect (SD)	P value^c^		
		Addictive	Dominant	Recessive		Addictive	Dominant	Recessive		Addictive	Dominant	Recessive
TG (mmol/l)	0.18 (0.35)	0.983	0.680	0.597	0.18 (0.09)	0.042*	0.112	0.079	0.21 (0.09)	0.021*	0.055	0.064
TC (mmol/l)	0.07 (0.13)	0.803	0.957	0.608	0.08 (0.13)	0.724	0.992	0.519	0.10 (0.13)	0.564	0.799	0.466
HDL-C (mmol/l)	0.02 (0.02)	0.252	0.399	0.292	0.04 (0.09)	0.789	0.997	0.628	0.07 (0.09)	0.499	0.663	0.488
LDL-C (mmol/l)	−0.04 (0.10)	0.858	0.953	0.695	−0.04 (0.04)	0.302	0.527	0.269	−0.03 (0.02)	0.128	0.266	0.159
Apoa1 (g/L)	0.02 (0.02)	0.437	0.530	0.517	0.02 (0.02)	0.356	0.418	0.485	0.01 (0.02)	0.646	0.726	0.683
Apob (g/L)	0.03 (0.03)	0.530	0.961	0.235	0.03 (0.02)	0.432	0.984	0.165	0.04 (0.03)	0.198	0.552	0.110

^a^Adujsted for age; ^b^Adjusted for age, income, smoking, drinking, physical activity and BMI; *TG* triglyceride, *TC* total cholesterol, *HDL-C* high-density lipoprotein cholesterol, *LDL-C* high low-density lipoprotein cholesterol, *Apoa1* Apolipoprotein A1, *Apob* Apolipoprotein B; ^c^Adjusted for age, income, smoking, drinking, physical activity, BMI, and gynoid fat mass percent; *P < 0.05

**Table 7:** The association between rs174547 and lipid profiles in women (*N* = 586)

Indices	Effect (SD)	P value^a^			Effect (SD)	P value^b^			Effect (SD)	P value^c^		
		Addictive	Dominant	Recessive		Addictive	Dominant	Recessive		Addictive	Dominant	Recessive
TG (mmol/l)	−0.09 (0.09)	0.541	0.888	0.344	−0.02 (0.09)	0.924	0.946	0.796	0.03 (0.08)	0.894	0.714	0.842
TC (mmol/l)	−0.03 (0.05)	0.513	0.607	0.564	−0.03 (0.08)	0.692	0.687	0.811	−0.01 (0.05)	0.822	0.828	0.881
HDL-C (mmol/l)	−0.04 (0.03)	0.155	0.119	0.469	−0.04 (0.06)	0.624	0.526	0.897	−0.02 (0.06)	0.817	0.724	0.996
LDL-C (mmol/l)	−0.04 (0.04)	0.386	0.426	0.531	−0.03 (0.02)	0.067	0.081	0.214	−0.04 (0.02)	0.035*	0.037*	0.183
Apoa1 (g/L)	0.02 (0.03)	0.754	0.891	0.460	0.01 (0.03)	0.989	0.789	0.764	−0.01 (0.03)	0.797	0.600	0.870
Apob (g/L)	−0.03 (0.02)	0.150	0.330	0.145	−0.01 (0.01)	0.320	0.424	0.392	−0.01 (0.02)	0.530	0.686	0.509

^a^Adujsted for age; ^b^Adjusted for age, income, smoking, drinking, physical activity and BMI; ^c^Adjusted for age, income, smoking, drinking, physical activity, BMI, and android fat mass percent; *TG* triglyceride, *TC* total cholesterol, *HDL-C* high-density lipoprotein cholesterol, *LDL-C* high low-density lipoprotein cholesterol, *Apoa1* Apolipoprotein A1, *Apob* Apolipoprotein B; *P < 0.05

## Discussion

In the present study, we found that the FA metabolism-related *FADS1* SNP rs174547 was significantly associated with several lipid metabolic indices and anthropometric indicators. We also confirmed an association between rs174547 and plasma phospholipid FA, and showed that rs174547 was associated with body fat distribution with a significant gender difference.

Several studies have previously explored the association of SNPs in the *FADS* gene cluster with plasma phospholipid FA in various ethnicities, and report the correlation of particular alleles with higher levels of LA and lower levels of AA [3, 7], which is consistent with our current results. Suhre et al. observed an association between D5D activity and rs174547 [4]; however, we demonstrated that rs174547 was more significantly associated with D6D. Similarly, the effect of rs174547 on dyslipidemia has been reported among many ethnic groups. The rs174547 C allele was previously found to be positively correlated with the level of TC and negatively correlated with the level of HDL-C in Chinese and other Asian populations [26–28]. However, Wu et al. found no significant associations of rs174547 with TG and TC levels among Chinese [19]. It has been acknowledged that the sexual dimorphism exists in general lipid profiles [29–32], so the association between the blood lipid indices and rs174547 might should be performed separately for female and male. Our results showed that rs174547 was significantly correlated with TC levels in men, and negatively correlated with LDL-C in women, which suggested that this locus may be a gender-specific SNP.

Gender differences in body fat distribution are well-established [14], with women known to predominantly accumulate subcutaneous fat in their limbs and hips, while men accumulate visceral fat in their abdomens; they therefore have an increased risk of obesity-related metabolic diseases [33, 34]. Body composition was precisely measured using DXA in our study, revealing that female C allele carriers had less abdominal fat reflected by the android FMP, and male C allele carriers had less subcutaneous fat reflected by the gynoid FMP. Thus, our findings indicated that FADS1 rs174547 might be a useful tool to elucidate the genetic basis of obesity-related disease, and it might be a predictive marker to identify high-risk individuals for abnormal body fat distribution and dyslipidemia, especially in male subjects. Besides, mutations of the same allele appeared to cause different body compositions among men and women, suggesting that gender differences in fat distribution reflect genetic diversity, which may involve differences in gene expression and function. It should be noted that the sexual differences in body fat distribution and lipid metabolism involve many genetic and environmental factors and their complex interactions. Our finding might partially explain this gender discrepancy.

A major strength of our study is that it is the first to demonstrate the impact of this FA-related SNP on gender differences in body fat distribution in a Chinese adult population. Although the influence of rs174547 on BMI has been studied previously [13], fat distribution measures derived from DXA are more precise and provide additional information to further understand the association with rs174547. We also thoroughly discussed the impact of gender differences on the association of rs174547 with blood lipid profiles. However, we acknowledge a number of limitations. First, we only focused on the effect of one SNP, and did not explore interaction effects between SNPs. Second, we did not consider the influence of environmental factors on genotype and phenotype, and most of the study participants were middle-aged adults. Finally, the study sample was from a capital city in eastern China and the subjects were not recruited through a random sampling procedure. In addition, the 951 sample size was relatively small. Thus our study sample might not be representative of the entire Chinese population. However, we focused on the association analysis between factors rather than descriptive analysis of the population in this study, the representativeness of the sample might not affect the association results. Further large and nationally representative studies were warranted to confirm the findings.

## Conclusions

In summary, we investigated the relationship between *FADS1* rs174547 with plasma phospholipid FA profiles, anthropometric measurements, and lipid profiles in this Chinese adult population. The rs174547 C allele was shown to correlate with the level of plasma FA. The association between the rs174547 genotype and body fat distribution and lipid indicators differed between men and women, which might partially explain sexual differences in body fat distribution and lipid metabolism. Future research should investigate the effects of *FADS1* and *FADS2* on body fat distribution, especially in women.

## Abbreviations

AA: Arachidonic acid
D5D: Δ5 desaturase
D6D: Δ6 desaturase
DGLA: Dihomo-γ-linolenic acid
DXA: Dual-energy X-ray absorptiometry
FA: Fatty acid
FADS: Fatty acid desaturase
FMP: Fat mass percentage
GLA: γ-linolenic acid
HDL-C: High-density lipoprotein cholesterol
HWE: Hardy–Weinberg equilibrium
LA: Linoleic acid
LDL-C: Low-density lipoprotein cholesterol
SNP: Single nucleotide polymorphism
TC: Total cholesterol
WC: Waist circumference
WHpR: Weight-to-hip ratio
