# Erratum to: A Single Nucleotide Polymorphism in the FADS1 Gene is Associated with Plasma Fatty Acid and Lipid Profiles and Might Explain Gender Difference in Body Fat Distribution

**DOI:** 10.1186/s12944-017-0479-5

**Published:** 2017-05-13

**Authors:** Huilan Guo, Lichao Zhang, Chaonan Zhu, Fei Yang, Shanshan Wang, Shankuan Zhu, Xiaoguang Ma

**Affiliations:** 1 Department of Nutrition and Food Hygiene, School of Public Health, School of Medicine, Zhejiang University, 866 Yu-hang-tang Road, Hangzhou, Zhejiang 310058 China; 2 Chronic Disease Research Institute, School of Public Health, School of Medicine, Zhejiang University, 866 Yu-hang-tang Road, Hangzhou, Zhejiang 310058 China; 3 Department of Public Health, Shandong Provincial Hospital affiliated to Shandong University, Jinan, Shandong China

## Erratum

After publication of the original article [1], it came to the authors’ attention that the Author contributions section contained an error. The contribution of Dr. Fei Yang is stated as follows: “YF carried out gas chromatography and assisted genotyping studies.”

In order to avoid any misunderstanding of the contribution of Dr. Fei Yang, the sentence should be updated to: “YF carried out gas chromatography data and assisted genotyping experiments.”

## References

[1] Guo H, Zhang L, Zhu C, Yang F, Wang S, Zhu S, Ma X (2017). A single nucleotide polymorphism in the FADS1 Gene is associated with plasma fatty acid and lipid profiles and might explain gender difference in body fat distribution. Lipids Health Dis.

